# Melatonin and its protective role in attenuating warm or cold hepatic ischaemia/reperfusion injury

**DOI:** 10.1111/cpr.13021

**Published:** 2021-03-10

**Authors:** Chenxia Hu, Lingfei Zhao, Fen Zhang, Lanjuan Li

**Affiliations:** ^1^ State Key Laboratory for the Diagnosis and Treatment of Infectious Diseases Collaborative Innovation Center for the Diagnosis and Treatment of Infectious Diseases The First Affiliated Hospital School of Medicine Zhejiang University Hangzhou China; ^2^ National Clinical Research Center for Infectious Diseases The First Affiliated Hospital School of Medicine Zhejiang University Hangzhou China; ^3^ Key Laboratory of Kidney Disease Prevention and Control Technology Kidney Disease Center Institute of Nephrology First Affiliated Hospital College of Medicine Zhejiang University Hangzhou China

**Keywords:** inflammation, injury, ischaemia/reperfusion, liver, melatonin, mitochondria

## Abstract

Although the liver is the only organ with regenerative capacity, various injury factors induce irreversible liver dysfunction and end‐stage liver disease. Liver resection and liver transplantation (LT) are effective treatments for individuals with liver failure, liver cirrhosis and liver cancers. The remnant or transplanted liver tissues will undergo hepatic ischaemia/reperfusion (IR), which leads to oxidative stress, inflammation, immune injury and liver damage. Moreover, systemic ischaemia induced by trauma, stroke, myocardial ischaemia, haemorrhagic shock and other injury factors also induces liver ischaemia/reperfusion injury (IRI) in individuals. Hepatic IRI can be divided into warm IRI, which is induced by liver surgery and systemic ischaemia, and cold IRI, which is induced by LT. Multiple studies have shown that melatonin (MT) acts as an endogenous free radical scavenger with antioxidant capacity and is also able to attenuate hepatic IRI via its anti‐inflammatory and antiapoptotic capacities. In this review, we discuss the potential mechanisms and current strategies of MT administration in liver surgery for protecting against warm or cold hepatic IRI. We highlight strategies to improve the efficacy and safety of MT for attenuating hepatic IRI in different conditions. After the potential mechanisms underlying the interactions between MT and other important cellular processes during hepatic IR are clarified, more opportunities will be available to use MT to treat liver diseases in the future.

## Graphical abstract

Melatonin serves as an endogenous free radical scavenger with antioxidation capacity, and it is also able to attenuate hepatic ischaemia/reperfusion injury via its anti‐inflammation, and antiapoptotic capacities.

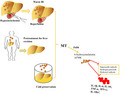

## 1 INTRODUCTION

Although the liver is the only organ with regenerative capacity, end‐stage liver dysfunction induces multiple organ failure and high mortality under extreme circumstances. Liver resection is an effective strategy to remove tumour tissue from patients with liver cancer, and liver transplantation (LT) is an effective treatment for individuals with liver failure, liver cirrhosis and liver cancers. The remnant liver or transplanted liver graft will undergo hepatic ischaemia/reperfusion (IR), which further leads to oxidative stress, inflammation, immune injury and liver damage.^1^ Liver ischaemia/reperfusion injury (IRI) also occurs in individuals with systemic ischaemia induced by trauma, stroke, myocardial ischaemia, haemorrhagic shock and other injury factors.^2^ Pre‐existing liver diseases not only directly lead to a high frequency of liver surgery but are also positively correlated with the severity of hepatic IRI.^3^ Moreover, obesity, older age and alcoholism can easily impair liver regenerative capacity and result in hepatic steatosis, which is associated with a higher complication index and postoperative mortality after liver surgery.^4^ Hepatic IRI can be divided into warm IRI, which is induced by liver surgery and systemic ischaemia, and cold IRI, which is induced by LT.^5^ Cessation of hepatic blood supply is always carried out by clamping manoeuvres and inevitably exposes the liver to IR and leads to liver dysfunction in mammals, while restoration of blood flow further exacerbates liver damage to ischaemic liver tissues.^6^ In LT, excised liver grafts are stored in cold preservation solutions before LT, which leads to cold IRI in transplants.^7^

Melatonin (MT), namely, N‐acetyl‐5‐methoxytryptamine, was first found to be synthesized from the amino acid tryptophan in the pineal gland and participates in the regulation of sleep promotion, circadian rhythms and neuroendocrine processes.^8^ In addition, MT participates in the regulation of energy metabolism, immune function, cardiovascular function, sexual behaviour, the neuropsychiatric system and reproduction.^9^, ^10^ It serves as a potent endogenous free radical scavenger that protects against mitochondrial damage and has beneficial effects on tissue injury by clearing reactive oxygen species (ROS) or reactive nitrogen species (RNS) in vitro and in vivo.^11^ In liver tissue, MT was reported to protect against oxidative damage and IRI via upregulation of glutathione (GSH) levels, maintenance of mitochondrial membrane structure and reduction of lipid peroxidation, oxidized glutathione (GSSG) levels and polymorphonuclear infiltration.^12^, ^13^ MT and its metabolites resist inflammation and prevent disturbances in mitochondrial redox reactions, biogenesis, dynamics and mitophagy to further protect against hepatic IRI.^14^, ^15^, ^16^

In this review, we discuss the potential mechanisms and current strategies of MT administration in liver surgery for protecting against warm or cold hepatic IRI. Although MT is considered an effective agent with protective effects against hepatic IR, we still highlight strategies to improve the efficacy and safety of MT for attenuating hepatic IRI in different conditions. After the potential mechanisms underlying the interaction between MT and other important cellular processes during hepatic IR are clarified, more opportunities will be available to use MT to treat clinical liver diseases in the future.

## 2 MITOCHONDRIAL DYSFUNCTION, INFLAMMATION AND IMMUNE RESPONSES IN HEPATIC IR

**FIGURE 1:**
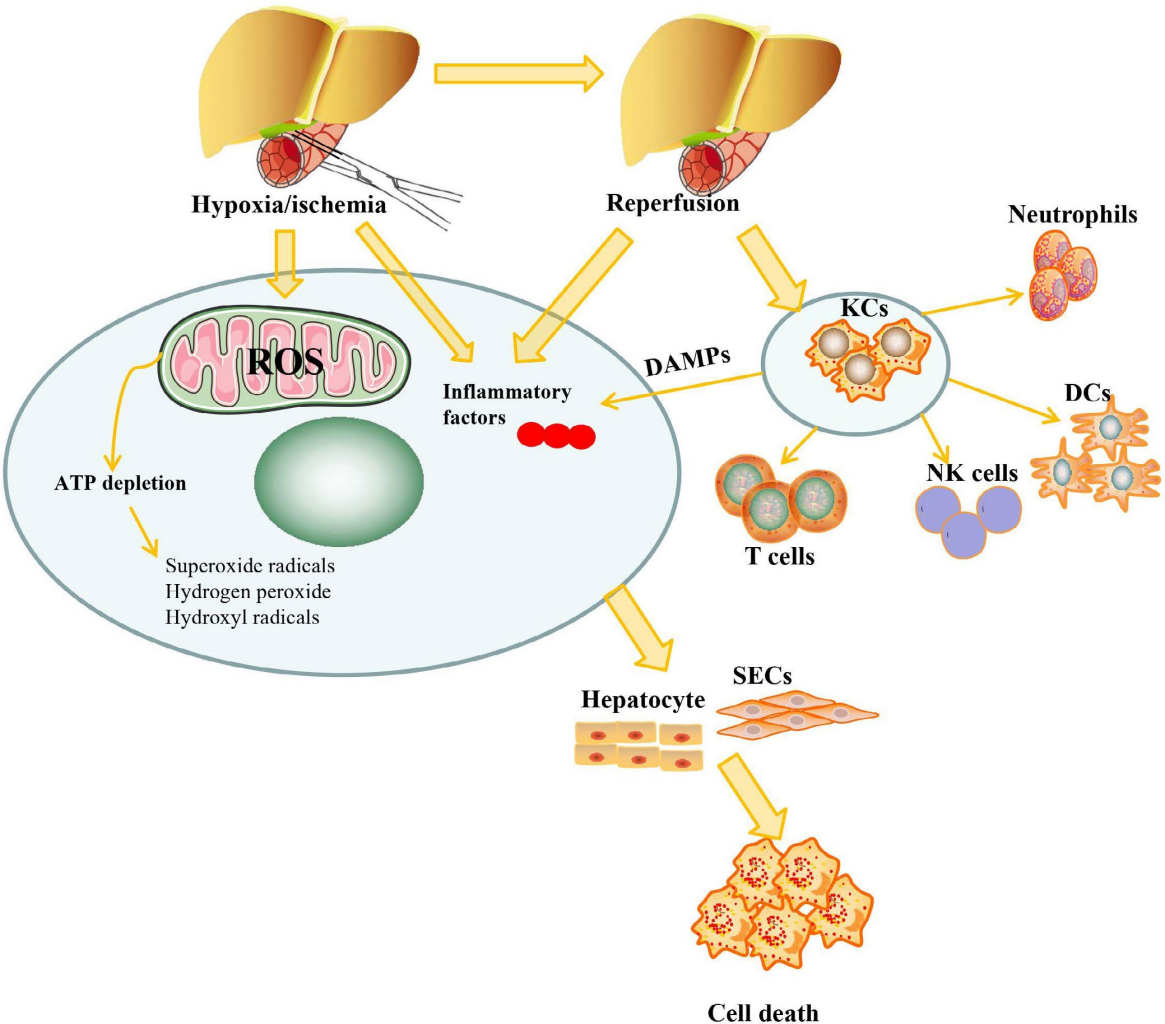
Hepatic ischaemia/reperfusion results in liver injury via activation of mitochondrial reactive oxygen species (ROS), inflammation and immune responses

Hepatic IR results in liver injury via activation of mitochondrial ROS, inflammation and immune responses (Figure 1). Depletion of blood flow in liver tissue transforms hepatocyte metabolism into anaerobic respiration and disrupts oxidative phosphorylation. Ischaemia limits the mitochondrial electron transport chain and further leads to deposition of electron carriers at the onset of reperfusion.^17^ A lack of oxygen supply results in parenchymal cell death as a consequence of metabolic disturbances, including glycogen consumption, adenosine triphosphate (ATP) depletion, xanthine oxidase conversion and intracellular pH reduction.^18^ Liver ischaemia also results in imbalances in Ca^2+^, H+ and Na+ homeostasis and mitochondrial depolarization and finally swelling of sinusoidal endothelial cells (SECs) and Kupffer cells (KCs).^19^ Reperfusion reintroduces oxygen into ischaemic tissue, and reperfusion injury is initiated by direct and indirect cytotoxic mechanisms such as oxidative stress injury, inflammation and immune cell recruitment.^20^ Oxidative stress promotes the generation of ATP metabolites accompanied by upregulation of superoxide radicals, hydrogen peroxide and hydroxyl radicals.^21^ The adaptive immune response and early and massive T‐cell recruitment were initiated after the preservation of ischaemic liver grafts in cold stock solution.^22^ Following reperfusion in LT recipients, liver grafts easily develop primary nonfunction or impaired primary function in which they undergo microcirculatory dysfunction and pH homeostasis disturbance.^23^, ^24^, ^25^ IR induces oxidative stress, which causes mitochondrial permeability transition pore (MPTP) opening, resulting in loss of mitochondrial membrane potential, mitochondrial swelling and decreased ATP generation.^26^ Oxidative stress further leads to the generation of ROS, inflammatory cytokines, and complement factors and the upregulation of autophagy, endoplasmic reticulum (ER) stress and mitochondrial dysfunction.^5^ Although mitochondrial or cytosolic ROS are unable to directly cause cytotoxicity in liver cells, they promote lipid peroxidation and stimulate the release of damage‐associated molecular patterns (DAMPs), namely, nuclear protein high‐mobility group box 1 (HMGB1). In response to Toll‐like receptor 4 (TLR4) on the surface of KCs, HMGB1 migrates from hepatocytes and binds to KCs to activate sterile inflammation and promote the generation of additional ROS.^27^ Lipid peroxidation further upregulates the release of cytochrome c into the cytoplasm, the activation of caspases and the initiation of cell death after the upregulation of mitochondrial membrane permeability and the loss of mitochondrial integrity.^28^

Reperfusion‐induced inflammation has been reported to induce hepatic IRI in parenchymal and nonparenchymal cells from in situ and transplanted liver grafts.^20^, ^29^ Various inflammatory cells, such as neutrophils, KCs, T lymphocytes, natural killer T (NKT) cells, and various humoral factors, such as complement factors, cytokines and chemokines, are activated to exacerbate injury in SECs and hepatocytes in hepatic IR models.^30^ KCs, which are the resident antigen‐presenting macrophages in liver tissue, can be first activated for the regulation of downstream inflammatory cells in circulation at the earliest stages of IR.^31^ KCs recognize circulating DAMPs to translate alarm signals into an overt inflammatory response. At the initial phase of reperfusion, KC activation enhances the generation of ROS^32^ and promotes the release of inflammatory cytokines such as tumour necrosis factor alpha (TNF‐α), interleukin (IL)‐1 and IL‐6. After that, IL‐1 and TNF‐α activate CD4^+^ T lymphocytes to generate TNF‐β, TNF‐γ and granulocyte‐macrophage colony‐stimulating factor (GM‐CSF).^33^ In contrast, the pathophysiology of hepatic IR is related to the level of nitric oxide (NO), which is produced by two synthase isoforms, endothelial NO synthase (eNOS) and inducible NO synthase (iNOS). Hepatic SECs constitutively and exclusively produce small amounts of NO to maintain endothelial function for short intervals, and eNOS contributes to the protective mechanism of the endothelium. However, inflammatory factors significantly increase the activity of iNOS to synthesize large amounts of NO and produce free radicals in hepatocytes, SECs, KCs and hepatic stellate cells (HSCs) for sustained periods.^34^ Circulating inflammatory factors also trigger the migration of nonresident neutrophils and monocytes into injured sites and initiate a second wave of ROS/RNS production that exacerbates hypoperfusion and the release of proteases, leading to hepatocyte toxicity.^35^, ^36^ At the late phase of perfusion, recruitment of neutrophils promotes the release of other inflammatory factors from injured sites,^32^ which leads to granulocyte accumulation in the sinusoidal space and microcirculatory disturbances.^37^

## 3 APOPTOSIS, NECROSIS AND AUTOPHAGY IN HEPATIC IR

Reduced oxidative phosphorylation results in failure of aerobic ATP formation and subsequent cell death of hepatocytes and SECs in anoxic and ischaemic microenvironments.^35^, ^38^ Several cell death modes, including apoptosis, necrosis and autophagy, can be found in liver grafts undergoing warm or cold IR.^39^ Mitochondrial dysfunction can induce two modes of cell death: apoptosis and necrosis. Mild injury induces hepatocyte apoptosis, while severe injury induces cell necrosis. Cell apoptosis is a kind of cell death characterized by cell shrinkage, chromatin condensation, nuclear fragmentation and the formation of apoptotic bodies. Necrosis is another kind of cell death characterized by mitochondrial and cell swelling, loss of plasma membrane integrity and vacuolization. Increased damage or intracellular ATP depletion converts the cell death mechanism from apoptosis to necrosis.^40^, ^41^ Mitochondria‐derived ROS increase lipid peroxidation and mitochondrial membrane permeability, ultimately leading to the release of cytochrome c and caspases and the exacerbation of apoptotic cell death.^28^ NO production and inflammatory cytokines also disrupt liver microcirculation and mitochondrial function and promote the generation of caspases, cytochrome c and antiapoptotic protein B‐cell lymphoma 2 (Bcl‐2) to promote hepatocyte necrosis and liver regeneration.^42^, ^43^, ^44^ In particular, three types of autophagy, macroautophagy, microautophagy and chaperone‐mediated autophagy (CMA), serve as self‐digestion methods to remove long‐lived proteins, damaged organelles and malformed proteins to supply ATP and nutrients in mammals.^45^, ^46^ Autophagy is reported to be regulated by different signalling pathways, such as the mammalian target of rapamycin (mTOR), mitogen‐activated protein kinase (MAPK), Bcl‐2 and p53 pathways.^47^ The mTOR signalling pathway is the main potent proinflammatory regulator that negatively regulates autophagy activation.^48^ Although autophagy can protect against hepatic IRI by counterbalancing ATP deprivation at the ischaemia stage, there is also evidence indicating that sustained and excessive activation of autophagy leads to the progression of cell death at the reperfusion phase.^49^, ^50^ The contradictory effects may be attributed to the fact that different types of IR and different liver conditions lead to different degrees of autophagy activation. To compensate for the lost liver function, all these pathophysiological changes convert injured livers to a regenerative state. Stathmin‐mediated mitosis was reported to be activated to promote hepatocyte proliferation around the perivascular regions after IR,^51^ and several nonparenchymal cells, such as KCs and HSCs, worked together to participate in liver tissue remodelling.^52^, ^53^ When regeneration is unable to counteract liver injury induced by excessive injury factors, irreversible liver damage will develop into acute or chronic liver failure. As the potential mechanisms underlying hepatic IRI are complex, targeting individual mechanisms makes it difficult to achieve the desired protection in attenuating hepatic IR‐induced liver injury.

## 4 THE METABOLIC AND SIGNALLING PATHWAYS OF MT IN LIVER TISSUE

**FIGURE 2:**
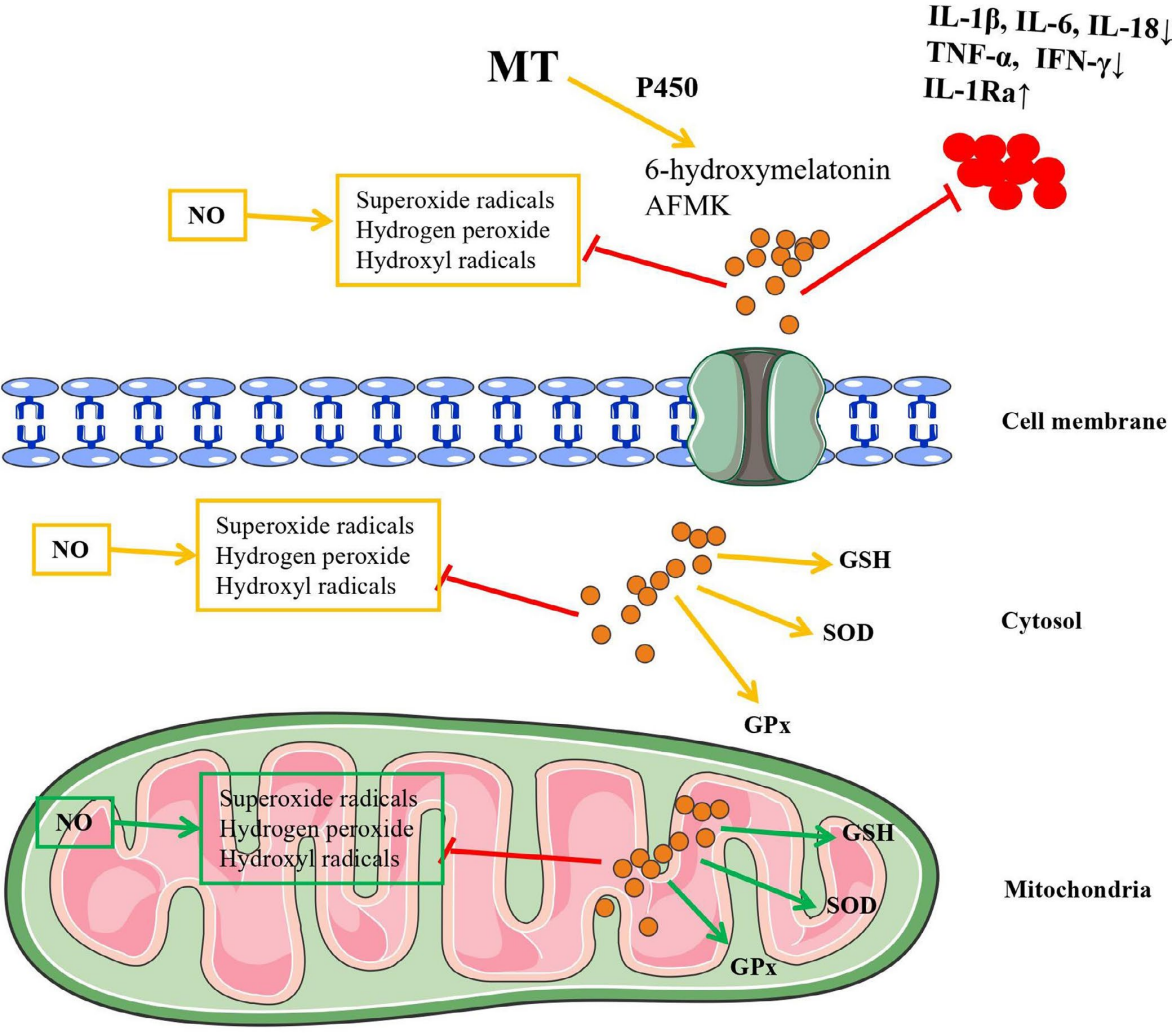
Melatonin (MT) and its metabolites protect against hepatic IRI by directly or indirectly inhibiting oxidative stress, inflammation and immune responses

The concentration of MT in liver tissue may depend on hepatic metabolic requirements, and this hormone is probably synthesized in the liver and intestinal tract.^54^, ^55^ The level of MT is generally highest in serum, while Lahiri et al documented that the second highest level of MT was in the liver.^56^ The gastrointestinal tract releases MT into the portal circulation and liver tissue; moreover, new data suggest that another source of MT is synthesis by the liver.^55^ MT is metabolized by cytochrome P450 in liver tissue and is transformed into 6‐hydroxymelatonin and N(1)‐acetyl‐N(2)‐formyl‐5‐methoxykynuramine (AFMK). MT and its metabolites protect against hepatic IRI by directly or indirectly inhibiting oxidative stress, inflammation and immune responses (Figure 2). However, severe oxidative stress promotes the nonenzymatic MT metabolism via its interaction with ROS and NOS.^57^ In the subcellular milieu of liver tissue, the MT concentration is highest in the cell membrane since the cell membrane acts as a reservoir of MT. Whenever MT is needed in other subcellular circumstances, MT is transferred from the cell membrane into the cell and from the cytosol to the mitochondria and nucleus. The transport mechanism in the subcellular milieu enables MT to have low toxicity when it is administered in high doses.^58^ Reiter and colleagues found that the concentration of MT in mitochondria isolated from rat hepatocytes greatly exceeded that in blood; moreover, pinealectomy did not decrease the level of MT in hepatocytes. This enables MT to act as a paracrine or autocrine factor to regulate intracellular events.^59^

Melatonin also protects against oxidative stress in liver IRI through multiple novel signalling pathways, such as the TLR, haem oxygenase‐1 (HO‐1) and c‐Jun N‐terminal kinase (JNK) pathways.^38^ Among the numerous pathways, crosstalk between MT and the TLR system in hepatic IR is pivotal. TLRs are pattern‐recognition receptors that recognize conserved pathogen‐associated molecular patterns (PAMPs). TLR4 is one of the putative HO‐1 repressors in noninfectious hepatic IR, which indicates that the crosstalk between HO‐1 and the TLR4 system is also important in hepatic IR.^60^ Kang and colleagues found that MT effectively inhibited the activation of JNK and extracellular signal‐regulated kinase (ERK) signalling, inhibited the nuclear translocation of nuclear transcription factors such as nuclear factor kB (NF‐κB) and c‐Jun, and induced the upregulation of HO‐1.^61^ HO‐1 is the rate‐limiting enzyme in haem degradation and has adaptive antioxidative and anti‐inflammatory effects in hepatic IRI. MT protected against liver IRI via upregulation of HO‐1 and suppression of the IFN signalling pathway downstream of TLR4. After that, MT augmented the level of NF‐E2‐related factor‐2 (Nrf2) nuclear translocation and attenuated the upregulation of Janus kinase 2 (JAK2) and signal transducer and activator of transcription 1 (STAT1).^62^ Dysregulation of JNK signalling has been suggested to be an important factor in the regulation of metabolism, inflammation and immune responses in the pathology of IRI.^63^ Administration of JNK inhibitors and their substrate c‐Jun significantly increased the survival rate of experimental models with liver resection and decreased the cell death rate of pericentral hepatocytes and nonparenchymal cells via activation of caspase‐3 and inhibition of cytochrome c release and lipid peroxidation.^64^

## 5 MT TREATMENTS PROTECT AGAINST HEPATIC IRI

Characterization of hepatic IR may unlock a novel therapeutic avenue in which ROS overexpression, and sterile inflammatory disorder can be targeted to preserve liver function during the progression of liver IR. MT was recently recognized as an effective factor in preserving liver function in liver grafts undergoing hepatic IR via its antioxidation, anti‐inflammation and antiapoptotic capacities (Table 1).

**TABLE 1:** MT treatment effectively improves the prognosis of liver resection and LT via different mechanisms

Animal	IR method	MT dose (mg/kg)	Route	Effect	Mechanism	Ref.
Rat	70 min ischaemia; 2 h reperfusion	10	Intraperitoneal	Protected against mitochondrial injury; decreased mitochondrial lipid peroxidation; increased mitochondrial glutathione peroxidase activity	Reduced mitochondrial oxidative stress; increased respiratory control index, State 3 respiration and dinitrophenol‐induced uncoupled respiration; improved hepatic mitochondrial energy transfer and energy metabolism	26
Rat	60 min ischaemia; 1 or 5 h reperfusion	10	Intraperitoneal	Reduced the level of serum aminotransferase; ameliorated IR‐induced liver damage	Decreased levels of TLR3 and TLR4; decreased serum level of HMGB1; suppressed the expression of MyD88, ERK, NF‐κB; suppressed phosphorylated JNK and phosphorylated c‐Jun; increased the level of TRIF expression; decreased the phosphorylation of IRF3 and IFN‐β; attenuated the levels of TNF‐α, IL‐6 and iNOS; increased the level of HO‐1	61
Rat	60 min ischaemia; 5 h reperfusion	10	Intraperitoneal	Decreased serum ALT activity	Increased HO activity; enhanced the level of Nrf2 nuclear translocation; decreased TLR4, TRIF, and MyD88; suppressed the interaction between TLR4/TRIF and TLR4/MyD88; decreased the levels of JAK2, STAT1 and IFN‐β	62
Rat	35 min ischaemia; 2, 4, 8 or 24 h reperfusion	10	Intraperitoneal	Downregulated the levels of ALT, AST and LDH	Decreased the level of MDA; increased the levels of SOD and GSH	65
Rat	60 min ischaemia; 5 h reperfusion	10	Intraperitoneal	Decreased serum ALT activity and lipid peroxidation; decreased the rate of mitochondrial swelling; decreased the release of cytochrome c and caspase‐3	Improved GSH content and mitochondrial glutamate dehydrogenase activity	66
Rat	60 min ischaemia; 2 h reperfusion	10	Intraperitoneal	Improved the survival rate; decreased ALT, AST and lipid peroxidation	Decreased plasma nitrite, TNF‐α, and iNOS expression; preserved the hepatic mitochondrial redox status	67
Rat	30 min ischaemia; reperfusion with nonischaemic liver tissue resection	50	Gavage	Improved animal survival rate; decreased transaminase levels; decreased cell necrosis; decreased liver damage	Decreased leucocyte infiltration and iNOS expression; inhibited the IKK and JNK pathways; improved cell proliferation	68
Rat	30 min ischaemia; 2 h reperfusion	20	Intraperitoneal	Decreased ALT, AST and LDH levels	Decreased serum levels of IL‐1β, IL‐6, IL‐18, TNF‐α and IFN‐ γ	69
Rat	35 min ischaemia; 2, 4, 8 or 24 h reperfusion	10	Intraperitoneal	Decreased necrosis	Decreased liver IL‐1β; increased IL‐1Ra	70
Mouse	1 h ischaemia; 3 h reperfusion	10	Intraperitoneal	Attenuated ALT and AST levels; ameliorated hepatic injury‐induced pathologic lesions	Increased the phosphorylation of Raf‐1, MEK1/2, and ERK1/2; increased the phosphorylation of p90RSK and Bad	71
Mouse	60 min ischaemia; 0, 1, 5 or 24 h reperfusion	10	Intraperitoneal	Decreased hepatocellular damage; decreased ALT and AST; decreased necrosis, sinusoidal congestion and hepatocyte vacuolization	Increased mTOR, 4E‐BP1 and 70S6K phosphorylation; decreased autophagic flux; decreased oxidative stress	72
Rat	30 min ischaemia; 6 h reperfusion	20	Intraperitoneal	Decreased the levels of ALT, AST, TNF‐α, MDA, liver injury index, and apoptotic index	Increased the levels of GPx and SOD	74
Rat	60 min ischaemia; 72 h reperfusion	20 or 50	Intraperitoneal	Decreased liver injury score and serum AST	Decreased the levels of inflammatory factors (TNF‐α, NF‐κB, IL‐1β, MMP‐9); decreased oxidative stress‐related factors (NOX‐1, NOX‐2, oxidized protein); decreased apoptotic factors (caspase‐3, PARP, Bax); decreased the release of cytosolic cytochrome c; suppressed mitochondrial permeability transition	75
Rat	60 min ischaemia; 72 h reperfusion	20 or 50	Intraperitoneal	Decreased liver injury score; decreased plasma AST level; inhibited DNA damage (γ‐H2AX) and mitochondrial damage (cytosolic cytochrome c)	Decreased the expression of inflammatory markers (ICAM‐1, IL‐1β, MMP‐9, TNF‐α, NF‐κB, RANTES); decreased the expression of apoptosis proteins (cleaved caspase‐3, PARP); decreased the expression of oxidative stress‐related factors (NOX‐1, NOX‐2)	76
Mouse	Reduced‐size LT	10	Intraperitoneal	Preserved liver function and promoted liver regeneration	Enhanced inflammatory Ly6C+ F4/80+ monocytes and promoted the release of IL‐6, IL‐10 and TNF‐α	87

## 6 MT TREATMENT OF WARM IR

Melatonin was reported to protect against IRI by improving mitochondrial respiration, ATP synthesis, mitochondrial swelling and lipid peroxidation in liver tissue.^26^ MT significantly decreased the malonaldehyde (MDA) level and increased the concentrations of superoxide dismutase (SOD) and GSH in vivo, subsequently downregulating the levels of alanine transaminase (ALT), aspartate transaminase (AST) and lactate dehydrogenase (LDH) in hepatic IR models.^65^ MT can not only decrease the level of lipid peroxidation but also improve mitochondrial glutamate dehydrogenase activity to decrease the rate of mitochondrial swelling and the release of cytochrome c and caspase‐3 in hepatic IR rats.^66^ Rodriguez‐Reynoso et al documented that exogenous MT effectively preserved liver function and energy metabolism in hepatic IR rats by inhibiting the generation of TNF‐α, iNOS and NO.^67^ MT also served as a hepatoprotective agent for the attenuation of warm liver IRI and improved animal survival time via inhibition of the IκB kinase (IKK) and JNK pathways. Consequently, MT significantly decreased iNOS expression and leucocyte infiltration to enhance cell proliferation and inhibit hepatocyte necrosis.^68^ MT pretreatment attenuated liver injury and preserved the levels of liver enzymes in animals with hepatic IR by downregulating serum levels of IL‐1β, IL‐6, IL‐18, TNF‐α and IFN‐γ but upregulating IL‐1Ra. ^69^, ^70^ MT was reported to upregulate the phosphorylation of p90RSK and Bad and downregulate the expression of cleaved caspase‐3 to protect against hepatic IRI.^71^ Furthermore, MT was shown to protect against liver IRI by downregulating autophagic flux and the phosphorylation of mTOR, 4E‐BP1 and 70S6K.^72^ Intriguingly, Nickkholgh et al studied 50 patients with liver resection and found that MT significantly decreased transaminase levels and shortened the stay time in the intensive care unit after operation without obvious side‐effects.^73^

To enhance the effects of MT in preserving liver function in hepatic IR models, different combinations of MT and other agents have been developed in current studies. Treatment with a combination of MT and dexamethasone more significantly improved liver function and decreased the tissue injury index and apoptosis rate of liver cells in hepatic IR animals than treatment with either agent alone. The underlying mechanism is that the combination of MT and dexamethasone increased the levels of glutathione peroxidase (GPx) and SOD but decreased the level of TNF‐α in vivo.^74^ Combined treatment with MT and exogenous mitochondria more significantly decreased the liver injury score and serum AST than treatment with MT or exogenous mitochondria alone in hepatic IR animals. MT significantly suppressed the mitochondrial permeability transition to inhibit the release of proteins related to inflammation, oxidative stress, apoptosis and mitochondrial damage.^75^ Likewise, post‐treatment with a combination of exosomes and MT more significantly improved the liver injury score and decreased the AST level by inhibiting the release of inflammatory markers, such as intercellular adhesion molecule (ICAM)‐1, IL‐1β, metalloprotease (MMP)‐9, TNF‐α, NF‐κB and regulated upon activation, normal T cell expressed and secreted (RANTES), and oxidative stress markers, such as nitrogen oxide (NOX)‐1 and NOX‐2. This combination also decreased the expression levels of apoptosis markers, including cleaved caspase‐3 and poly‐ADP‐ribose polymerase (PARP), a DNA damage marker (γ‐H2AX) and a mitochondrial damage marker (cytochrome c), to protect against hepatic IR injury.^76^

## 7 MT TREATMENT OF COLD IR

**FIGURE 3:**
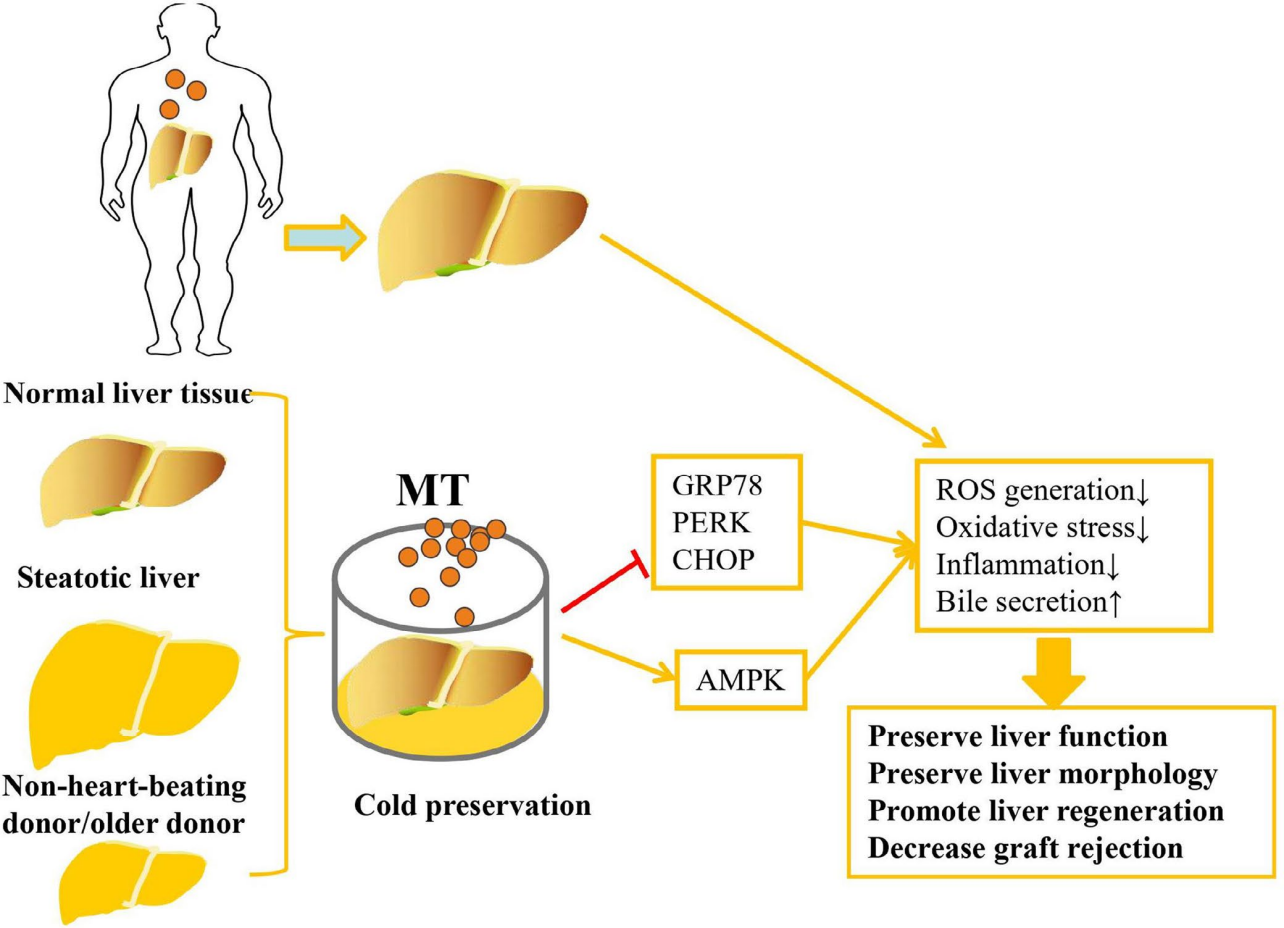
Addition of melatonin (MT) to the preservation solution or administration of MT to liver donors significantly improves liver function and inhibits graft rejection in liver transplantation

In individuals who undergo LT, the high incidences of graft rejection and early transplant failure in LT significantly increase the need for liver retransplantation.^77^ As LT is limited by restricted liver donor sources, doctors have expanded to performing surgery with marginal liver grafts from nonheart‐beating donors, older donors and steatotic liver tissues. These grafts are more vulnerable to cold IRI.^78^ Excised liver grafts are stored in preservation solutions, and some components guarantee their effectiveness, while other components have a marginal effect on liver grafts. The most common solution used to store liver grafts is University of Wisconsin (UW) solution. The storage of liver grafts is limited to 12 hours by simple hypothermia, and UW solution has various disadvantages, such as high viscosity and high concentration of K^+^.^79^ Institute George Lopez (IGL‐1) solution was developed based on UW solution to attenuate the risk of cardiovascular complications and provide greater protection of fatty livers via attenuation of oxidative stress and mitochondrial damage.^80^ To improve the function of isolated liver grafts, effective agents such as MT were added to the preservation solution or administered to liver donors (Figure 3). Cold‐preserved livers perfused without MT showed a narrow portal region and lower ATP content, while MT preserved liver morphology and decreased ROS generation in liver tissues that underwent cold IR.^81^ Furthermore, MT preserved the liver function of excursive nonsteatotic and steatotic liver tissue, accompanied by lower transaminase levels and higher bile production and sulphobromophthalein (BSP) clearance. MT improved bile secretion but decreased the r‐glutamyl transpeptidase level, subsequently significantly improving the restoration of liver function after cold storage and reperfusion in liver grafts in a dose‐dependent manner.^82^ MT was suggested to improve NO generation and inhibit oxidative stress and inflammation in liver grafts to protect against cold IR.^83^ Although steatotic livers reduce the shortage of donors for LT, they exhibit exacerbated ER stress and increase the incidence of cold IRI and graft dysfunction. The addition of MT to the preservation solution significantly attenuated hepatic injury and improved bile production in steatotic livers by decreasing the activities of glucose‐regulated protein 78 kDa (GRP78), protein kinase‐like endoplasmic reticulum kinase (PERK), and CHOP and upregulating autophagic marker expression and adenosine monophosphate‐activated protein kinase (AMPK) phosphorylation.^84^ In addition, a cocktail consisting of MT and other drugs (pentoxifylline, glycine, deferoxamine, N‐acetylcysteine, erythropoietin and simvastatin) provided effective protection against IR in steatotic liver grafts via attenuation of hepatic leucocyte infiltration, vacuolization and cell death. This multidrug treatment also decreased the levels of TNF‐α and ICAM‐1 and restored liver function to nearly the control level.^85^ Another multidrug cocktail based on MT, curcumin, simvastatin, N‐acetylcysteine, erythropoietin, pentoxyphylline, glycine and methylprednisolone for donor preconditioning significantly improved cell membrane integrity and decreased the levels of ALT, AST and LDH in excursive liver grafts. Moreover, the multidrug pretreatment also restored bile flow and reduced the levels of TNF‐α, IL‐6 and MDA in the grafts.^86^ In a model with reduced‐size LT, MT preserved liver function and promoted liver regeneration by enhancing inflammatory Ly6C+ F4/80+ monocytes and promoting the release of IL‐6, IL‐10 and TNF‐α.^87^ Intriguingly, MT also had beneficial effects on patients with hepatocellular carcinoma (HCC) who underwent LT. MT increased the activity of antioxidants but decreased the level of MDA to improve their survival rate.^88^

## 8 CONCLUSION

Warm and cold IRI in liver tissue are mediated by various mechanisms, such as mitochondrial dysfunction, liver microcirculation dysfunction and inflammation. Ischaemia initiates the generation of excessive free radicals in the liver, while reperfusion exacerbates liver injury after impairing the respiratory chain, metabolic enzymes and mitochondrial membrane structure. Consequently, IR leads to mitochondrial dysfunction, ATP depletion and the release of apoptotic factors in remnant or cold‐preserved liver grafts. Although activation of autophagy serves as a protective mechanism following warm or cold IR by removing damaged intracellular contents and maintaining ATP production, excessive upregulation of autophagy destroys essential proteins and organelles and leads to cell death in liver tissue during the severe ischaemic period. Endogenous MT is able to decrease inflammatory factors and maintain the mitochondrial redox state and mitochondrial biogenesis, consequently attenuating hepatic IRI in liver resection and LT. The evidence compiled in this review will serve as a comprehensive reference for the actions of MT in liver IRI and hopefully aid in the design of future research. MT is considered an effective agent with protective effects against hepatic IR, and there is currently a major shortage of studies on the application of MT in LT recipients. Furthermore, we highlighted strategies to improve the efficacy and safety of MT for attenuating hepatic IRI in different conditions. After the potential mechanisms underlying the interaction between MT and other important cellular processes during hepatic IR are clarified, more opportunities will be available to use MT to treat liver diseases in the future.

## CONFLICTS OF INTEREST

The authors declare that they have no conflicts of interest.

## AUTHOR CONTRIBUTIONS

Lanjuan Li contributed to the conception of this study. Chenxia Hu and Lingfei Zhao drafted the manuscript. Chenxia Hu and Fen Zhang revised the manuscript. All authors read and approved the final manuscript.

### PEER REVIEW

The peer review history for this article is available at https://publons.com/publon/10.1111/cpr.13021.

## Data Availability

Data sharing is not applicable to this article as no new data were created or analysed in this study.
